# Does Atmospheric Corrosion Alter the Sound Quality of the Bronze Used for Manufacturing Bells?

**DOI:** 10.3390/ma16134763

**Published:** 2023-06-30

**Authors:** Mourad Bentahar, Aline Petitmangin, Caroline Blanc, Anne Chabas, Silvio Montresor, Christophe Niclaeys, Ahmed Elbartali, Denis Najjar, Romain Duccini, Mathieu Jean, Sophie Nowak, Rémy Pires-Brazuna, Pierre Dubot

**Affiliations:** 1 Laboratoire d’Acoustique de l’Université du Mans (LAUM), UMR CNRS 6613, Institut d’Acoustique-Graduate School (IA-GS), CNRS, Le Mans Université, 72085 Le Mans, France; mourad.bentahar@univ-lemans.fr (M.B.); silvio.montresor@univ-lemans.fr (S.M.); romain.duccini.etu@univ-lemans.fr (R.D.); mathieu.jean.etu@univ-lemans.fr (M.J.); 2 Université Paris Est Creteil and Université de Paris, CNRS, LISA, F-94010 Créteil, France; caroline.blanc@lisa.ipsl.fr (C.B.); anne.chabas@lisa.ipsl.fr (A.C.); 3 UMR 9013-LaMcube-Laboratoire de Mécanique Multiphysique Multiéchelle, University Lille, CNRS, Centrale Lille, F-59000 Lille, France; christophe.niclaeys@centralelille.fr (C.N.); ahmed.elbartali@centralelille.fr (A.E.); denis.najjar@centralelille.fr (D.N.); 4 Université de Paris, CNRS, ITODYS, F-75006 Paris, France; sophie.nowak@u-paris.fr; 5 Université Paris Est Creteil, CNRS, ICMPE, UMR 7182, 2 Rue Henri Dunant, 94320 Thiais, France; remy.pires-brazuna@cnrs.fr (R.P.-B.); pierre.dubot@cnrs.fr (P.D.)

**Keywords:** Cu-Sn alloy, atmospheric chamber, materials characterization, resonance, nonlinear vibration, damping

## Abstract

Bells are made of bronze, an alloy of copper and tin. Art objects and musical instruments belong to tangible and intangible heritage. The effect of atmospheric alteration on their sound is not well documented. To address this question, alteration cycles of bronze specimens are performed in a chamber reproducing a realistic polluted coastal atmosphere. The corrosion layers are characterized by X-ray diffraction, electron microscopy and X-ray photoelectron spectrometry. The buried interface of the film (alloy-layer interface) is formed by a thin, adherent and micro-cracked layer, mainly composed of sulfates, copper oxide and chloride, on top of tin corrosion products. Near the atmosphere-film interface, less adherent irregular clusters of soot, calcite, gypsum and halite developed. Through these observations, an alteration scenario is proposed. To correlate the bronze corrosion effect on the bell sound, linear and nonlinear resonance experiments are performed on the corroded bronze specimens, where resonance parameters are monitored as a function of increasing driving force using a shaker. Results show that the corrosion effect on the acoustic properties can be monitored through the evolution of the acoustic nonlinear parameters (damping and resonance). These well-calibrated original experiments confirm the effect of corrosion on the acoustic properties of bronze.

## 1. Introduction

Bronzes are Cu-Sn alloys with different structures, depending on their tin content [1,2,3]. Under 15 weight percent (wt%) of tin, a predominant dendritic α-phase structure and traces of δ-phase areas, in the form of (α + δ) eutectoid between the dendritic arms in the alloy, are observed. For tin contents between 15 and 27 wt%, the (α + δ) eutectoid increases. Over 26 wt%, fine δ dendrites surrounded by the (α + δ) eutectoid are observed. The Cu-Sn alloys corrode when in contact with the atmosphere. Their corrosion is due to ionic migration occurring through the corrosion layer [4], which has developed and grown over time, forming a diffusion barrier for the electrolyte liquid layer that can appear on the metal surface during rain or water condensation episodes. The interactions among oxygen, alternating wet/dry cycles, gaseous pollutants, such as SO_2_, O_3_ and NO_2_ [5,6], and hydrophilic particles [7,8], can lead to two different corrosion mechanisms [4]. In less corrosive environments, Type I corrosion with a slow rate of alteration is governed by the diffusion of cations from the alloy to the surface. A Sn-rich layer in contact with the alloy passivates the object. Oxidized copper species diffuse through this Sn-rich layer and react with anions to form insoluble copper (II) products or dissolve in the environment. The contour and volume of the object are maintained. In more aggressive environments, a Type II mechanism organizes and installs anionic control that governs the higher rate of corrosion. In sheltered conditions, thick, porous corrosion layers cover a Sn-rich layer. Rain gradually leaches out the copper and dissolves the bronze. In the Type II mechanism, the volume and contour of the object are altered.

Exposed in steeples or campaniles, the bronzes used in bell manufacturing are α/δ alloys with a high tin content (22–25 wt%) [9] that are also submitted to atmospheric corrosion. Considered as valuable decorative works of art, musical instruments and efficient ways of communication, the bells combine the characteristics of tangible and intangible heritage that must be preserved. Among the most famous bell bronzes, one can mention Big Ben in the United Kingdom (1858), the bell of the Yongle regime in Pekin (1420) but also the bells of “Notre Dame de Paris” in France whose replacement in 2013 was widely publicized. The corrosion of bell bronzes has been poorly studied, with the exception of [10,11] that show that the long-term corrosion of the bell is governed by micro-infiltrating net-works of environmental fluids through brittle corrosion products that lead to a different α/δ corrosion behavior: a multilayer corrosion of the α-phase, while the corroded δ-phase is enriched in tin oxide. However, a question arises: can the atmospheric alteration also change the bronze vibration modes? This subject has not been studied in the literature. Therefore, the aim of this article is to investigate the potential impact of the atmosphere on the sound properties of bell bronze.

Bronze specimens were cast and then corroded in an experimental chamber. They were exposed to a polluted marine environment known for its corrosive properties [12,13]. Non-destructive (electron and interferometric microscopy) and microdestructive (XPS spectroscopy) techniques were used to study their physicochemical alterations. To evaluate the effects of this atmospheric corrosion on the dynamic vibration (resonance) behavior of the bronze, the resonance frequencies of the corroded specimens and their damping (or quality factor) were evaluated under linear and nonlinear vibration conditions. Nonlinear techniques are known for their sensitivity to the mesoscopic structural evolutions of different materials (composites, metals, concrete…) [14,15,16,17]. Through excited standing waves, the evolution of real and imaginary components of the elastic modulus can be evaluated through the excited resonances. The present study has the advantage to be more focused on the cases where corrosion is mainly localized on the surface of the samples studied, contrary to the experiences frequently described in the literature. Indeed, the bulk of the samples remains intact as well as the opposite side.

The aim of the present contribution is first to create and characterize atmospheric corrosion reproduced under laboratory conditions before detecting, with the help of the acoustic method, the impact of the created corrosion on the acoustic properties of the bronze, which is used to manufacture bells.

## 2. Materials and Methods

### 2.1. Bronze Samples

A bronze block of 130 mm × 88 mm × 30 mm was sand cast by the Centre Technique des Industries de la Fonderie (CTIF) in Sèvres (France). A polyurethane resin (Pontex resin) was used as a binding agent. This work considered 17 parallelepiped specimens: 11 specimens of size 120 mm × 12.5 mm × 5 mm and 6 smaller specimens of size 20 mm × 12.5 mm × 5 mm All samples were polished with ethanol using SiC paper and diamond paste: SIC 4000 grain (10 µm) on 5 faces and mirror polished (1 µm) on the 6th face measuring 120 mm × 12.5 mm and 20 mm × 12.5 mm, respectively. The 6 samples of 20 mm × 12.5 mm × 5 mm were corroded in the CIME chamber (2 samples per cycle) and used for the physicochemical analysis of the alteration. The 20 mm × 12.5 mm face, which has a more uniform initial surface condition, was chosen as a reference to evaluate the physicochemical properties of the weathering reproduced in the laboratory. A surface and cross-sectional analysis of the corrosion layer was undertaken. To perform the cross-section, the corroded samples were cast in resin to best preserve the integrity of the corrosion layer, and then cut and polished using SIC paper and diamond paste with ethanol to avoid any phase changes due to water contact. Regarding acoustic measurements, for repeatability, five uncorroded specimens (120 mm × 12.5 mm × 5 mm) were used as a reference to determine the acoustic properties of the unaltered bell bronze, and the remaining six samples were corroded for three cycles (2 specimens per cycle) in the CIME chamber. To ensure reproducibility, acoustic analysis was performed by keeping the same position for each specimen, which considers the uniform initial surface (120 mm × 12.5 mm) during the measurement.

The chemical compositions of 5 uncorroded bronze samples (8 mm^3^ each), taken at different depths in the block, were analyzed by Inductively Coupled Plasma—Optical Emission Spectrometry (ICP-OES, VARIAN Vista-Pro (Palo Alto, CA, USA)) according to the protocol described in [10]. There were no significant compositional differences between the alloys of the various sampling areas. Therefore, the average composition of all the samples 1–5 (Table 1) can be considered as being representative of the alloy.

**Figure 1:**
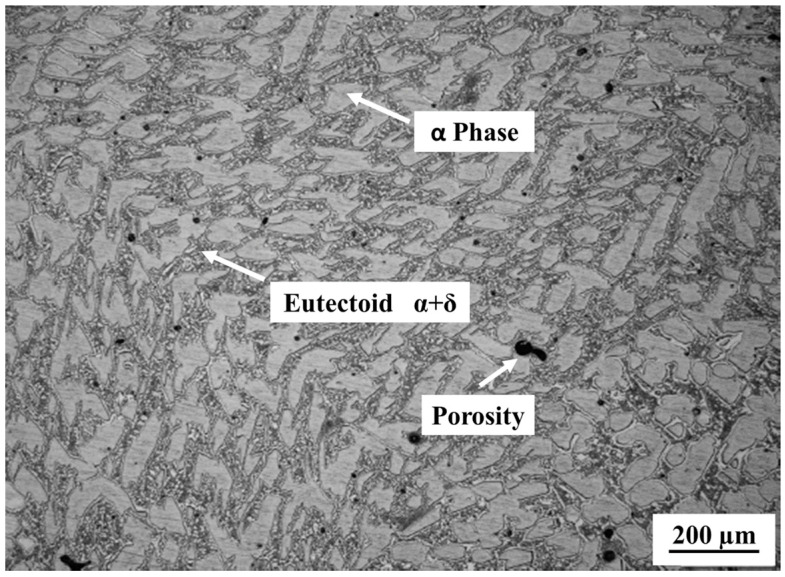
Optical micrography of the microstructure of the alloy.

The average content of copper (78.25% by weight) and tin (21.74% by weight) is characteristic of bronze bells [10]. Some traces of other metals are observed, but in proportions (<0.03 wt%) too low to alter the tonal properties of the alloy. Indeed, only Pb or Zn contents exceeding 1.5%wt can have a negative impact on the sound duration [9]. The specimens have a characteristic bell bronze microstructure: a dendritic α phase with an α/δ eutectoid constituent intercalated between the arms of the dendrites (Figure 1). Very few micro-cracks and porosities, which could influence the sound properties of the bronze [18,19,20], are present in the alloy.

### 2.2. Alteration in the CIME Chamber

The Chamber of Interaction between the Materials and the Environment (CIME) is a chamber designed in the laboratory to reproduce the impact of gaseous and particulate pollutants on materials under controlled temperature and humidity conditions. A detailed technical description of CIME is given in [21]. Briefly, a stainless-steel tank is connected to generators and analyzers dedicated to gases (SO_2_, NO_2_, CO_2_, O_3_ and VOC) and atmospheric particles of natural or anthropic origin. Temperature, humidity and pressure sensors are used to control microclimatic conditions.

In this study, a polluted coastal atmosphere was chosen to favor the development of weathering on bronze specimens [22]. The coastal atmosphere was reproduced by atomizing a NaCl-enriched solution. The urban atmosphere contains primary (SO_2_) and secondary (O_3_) gaseous pollutants and combustion particles (soot) from transportation and terrigenous particles from the remobilization of soil dust (calcite) [23]. The mean temperature of 20 °C (±0.1%) and relative humidity of 97% (±3%) optimize the metal corrosion. The concentrations of 400 ppb SO_2_ and 600 ppb O_3_ injected into the CIME represent 4 times the annual maximum measured by the Ile de France (region of Paris) air quality monitoring network (Airparif) over the last 25 years. Compared with conventional corrosion chambers, which reproduce pollutant concentrations in the % or ppm range, CIME offers weathering conditions that are closer to real environmental conditions.

A weathering cycle consists of successive injections of gas (15 days, 3 times a day), particles (3 days, 1 day per type of dust) and gas (15 days, 3 times a day). Under these experimental conditions, gaseous pollutants react with bronze coupons and relative humidity to form copper and tin oxides, sulfides and sulfates [21]. These products, with their hygroscopic properties, form a rough layer that makes it easier for the particles to adhere. This adhesion is reinforced by the second injection of gas, which also reacts with the deposited particles [24,25].

### 2.3. Physicochemical Characterization of the Corroded Bronze Specimens

The crystalline phases formed during the physicochemical alteration of bronze were identified by X-ray diffraction (Panalytical Empyrean, Palaiseau, France) with Cu radiation (λ_Kα_ = 1.541874 Å). Under θ–θ conditions, the pattern was recorded with a step size of 0.007° (300 s per step) and in the range of 10°–70°. At grazing incidence, a 3° incidence angle was applied with a step size of 0.009° (220 s per step) in the same range. The interest in grazing incidence X-ray diffraction was mainly to allow the analysis of the extreme corroded surface. Indeed, the penetrating depth of the grazing incidence is limited to 100 nm (as compared to approximately 1 µm for the Bragg-Brentano configuration).

The roughness of the potentially brittle alteration layer was measured by 3D interferometric microscope (NT1100 VEECO, Plainview, NY, USA), which is a non-destructive technique. In agreement with the ISO 25178-2:2021 standard, the 3D surface roughness parameter (Sz) was adopted to quantify the maximum height of the corroded surface (the difference between the highest points and the deepest valley). This parameter is also representative of its complexity. Sz values were averaged from at least 40 analyzes of 0.9 × 1.2 mm^2^.

The morphology and composition of the patina were observed on the surface and in sections by Scanning Electron Microscopy (SEM) with a Carl Zeiss MERLIN operating at 15 kV (Oberkochen, Germany) and analyzed by Energy Dispersive X-ray Spectroscopy (EDS) thanks to a 50 mm^2^ X-Max Silicon Drift Detector (SDD) from Oxford Instruments coupled with an AZtec operating system (Abingdon-on-Thames, UK). The samples were previously coated with a conductive layer of platinum by sputtering thanks to a Cressing-ton 208 HR sputter-coater monitored with a thickness controller Cressington MTM 20 (Watford, UK).

To qualitatively determine the film composition and oxide distribution in the patina, X-ray Photoelectron Spectroscopy (XPS) analysis was performed using a Thermo Scientific K-Alpha XPS instrument (Waltham, MA, USA) equipped with a monochromatic Al X-ray source (1486.6 eV, spot size 400 μm). The sample was abraded with an argon beam. First, a soft sputtering (kinetic energy of 200 eV, a speed of 1 Å/s and a stripping time of 10 s) was performed to remove surface contamination and analyze the topmost surface layer. Then, several sputterings (kinetic energy of 3000 eV, a speed of 1 nm/s and a stripping time of 30 s) were performed to study the in-depth patina composition in detail. The pressure never exceeded 5 × 10^−9^ mbar in the analysis chamber. XPS- C 1s, Ca 2p S 2p, Cl 2p O 1s, Cu 2p and Sn 3d spectra as well as AES-Cu LVV and AES-Na KLL spectra were studied. All spectra were energetically referenced to the C 1s line of the hydrocarbon contaminant (285.0 eV).

### 2.4. Acoustic Characterization of the Corroded Bronze Specimens with Nonlinear Resonances

#### 2.4.1. Experimental Device and Identification of the Resonance Modes

**Figure 2:**
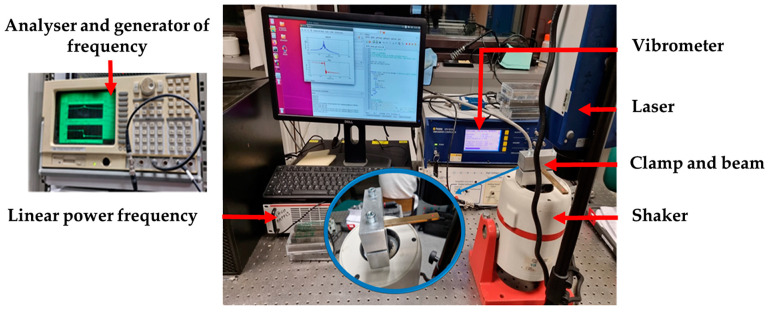
Experimental set-up to monitor the dynamic response of the bronze beams.

The dynamic characterization, at resonance, of the bronze beams in the intact and corroded states is carried out using the experimental setup shown in Figure 2.

**Figure 3:**
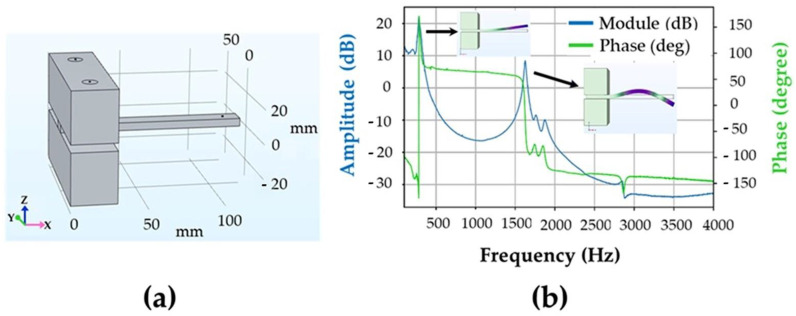
(**a**) Simulation conditions and (**b**) simulated resonance curves of the intact sample corresponding to 1st and 2nd order bending modes.

Numerical simulations are made using the software COMSOL Multiphysics^TM^ (version 6.1) to identify the generated resonance modes. Finite element simulations are based on the mechanical properties of the bronze that have been obtained experimentally with the help of ultrasonic measurements. Density, Poisson ratio and Young modulus have been found as $\rho =8798\;kg/m^{3}$; ν=0.335; E=92 GPa, respectively. Simulated resonance curves are presented in Figure 3.

#### 2.4.2. Nonlinear Resonance Measurements

In general, acoustic nonlinear measurements probe second-order effects related to the evolution of the complex elastic modulus as a function of the excitation level [26,27]. The elastic modulus can be written as:

(1) $$K\left(\varepsilon ,\dot{\varepsilon }\right)=K_{0}\left(1+\beta \varepsilon \left(t\right)+\delta {\varepsilon \left(t\right)}^{2}+\cdots \right)+\;H\left(\varepsilon ,sign\left(\dot{\varepsilon }\right)\right)$$

where $K_{0}$ is the linear modulus (at low dynamic strains), ε is the strain, β and δ represent the classical quadratic and cubic nonlinear parameters, respectively. ∆ε is the local strain amplitude over the previous period and $\dot{\varepsilon }$ is the strain rate. The function $H(\varepsilon ,sign\left(\dot{\varepsilon }\right))$ takes into account the hysteresis in the stress-strain relationship. $Sign\left(\dot{\varepsilon }\right)=1$ when $\dot{\varepsilon }>0$ and $sign\left(\dot{\varepsilon }\right)=-1$ when $\dot{\varepsilon }<0$ [27,28].

**Figure 4:**
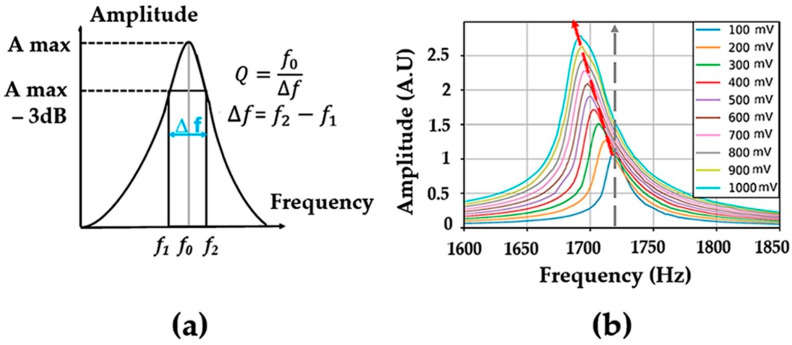
(**a**) Determination of the quality factor from a resonance curve; (**b**) Evolution of the bending resonance as a function of the excitation level for a bronze sample taken at the corroded state. The vertical line corresponds to the linear resonance (weak excitation).

When modeling resonance experiments, a decrease in the resonance frequency and the quality factor can be observed for increasing excitations:

(2) $$\frac{f_{0}-f}{f_{0}}=C_{1}\varepsilon .\;\frac{1}{Q}-\frac{1}{Q_{0}}=C_{2}\varepsilon .$$

where $Q_{0}$ and Q are the quality factors measured at low ($\varepsilon_{0}$) and higher (ε) strain amplitudes. f is the resonance frequency at strain amplitude ε, $f_{0}$ is the resonance frequency at low strain amplitude (or excitation) $\varepsilon_{0}$. $C_{1}$ and $C_{2}$ represent the hysteretic nonlinearity corresponding to frequency $\left(\alpha_{f}\right)$ and quality factor $\left(\alpha_{Q}\right)$ [29]. The abovementioned coefficients were determined for different materials and were found to be very sensitive to the changes existing in their microstructures [17,30]. Figure 4 shows the evolution of the bending resonance as a function of the excitation level. The observed decrease in the resonance frequency is a consequence of the decrease in the elastic modulus (known as softening) due to the existing soft regions which are mainly situated in the corroded and micro-cracked areas.

## 3. Results

### 3.1. Effect of the Corrosion on the Physicochemical Properties of Bronze Samples

#### 3.1.1. Characterization of the Surface

**Figure 5:**
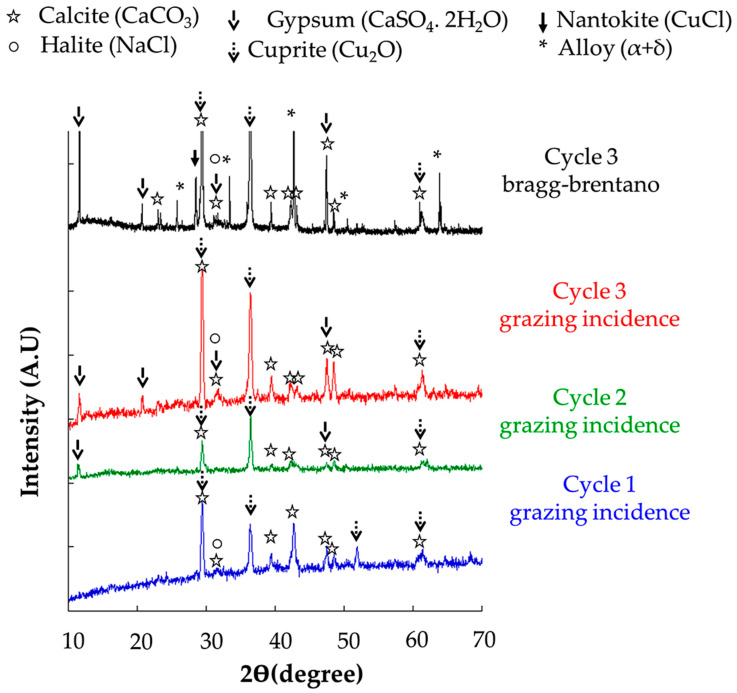
XRD spectra of the alteration layers in grazing incidence (cycles 1, 2, 3) and Bragg-Brentano (cycle 3) configuration.

To document the evolution of crystallization on the outer surface, XRD spectra obtained under grazing incidence after one, two, and three cycles are presented in Figure 5. Whatever the duration of the alteration, calcite and halite are detected. Because of their amorphous character, soot particles cannot be observed by XRD. The two main peaks of gypsum CaSO_4_·2H_2_O (11.65° and 20.74°) appear from the second cycle and increase in intensity afterwards. Concerning the corrosion products of the alloy, the crystallization of cuprite Cu_2_O (main peak at 36.5°) is observed from the first cycle and develops with the duration of the alteration.

Whatever the duration of corrosion, an increase in the intensity of the main gypsum peak and a new phase, the nantokite CuCl, are observed (note that only the results of Cycle 3 are presented in Figure 5) in a Bragg Brentano configuration. However, nantokite is not detected under grazing incidence. This result suggests a deeper development of the nantokite in the corrosion layer. This point will be studied in detail in Section 3.1.2 and discussed in Section 4.

**Figure 6:**
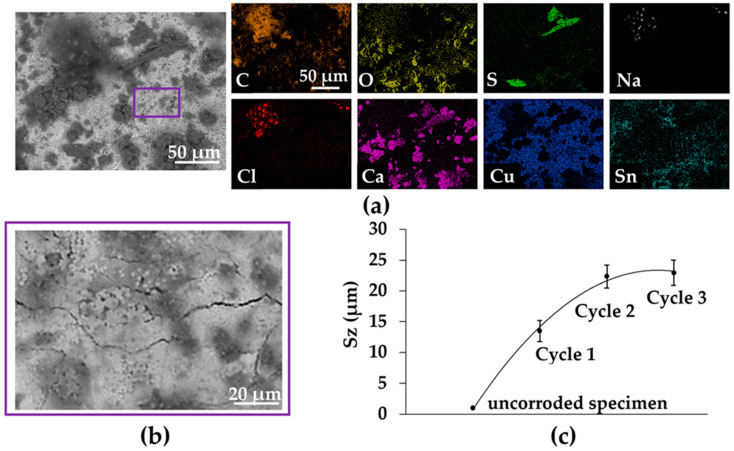
(**a**) SEM image and EDS analysis of the alteration layer following the cycle 3; (**b**) SEM image of the microcracked zone surrounded (within the purple frame); (**c**) Roughness of the alteration surface at uncorroded state and different cycles of corrosion.

On the other hand, SEM-EDS analyzes of the corroded surface were also performed (Figure 6a). The signal corresponding to oxygen is present all over the altered surface. The one for sulfur is more contrasted with a diffuse signal associated with copper and oxygen and a more localized and higher signal with Ca, O and C, which is characteristic of the initial calcite deposit. Thus, the CaCO_3_ particles appear as preferential reaction sites for SO_2_, leading to the crystallization of CaSO_4_·2H_2_O gypsum under orthorhombic rods as seen in Figure 6a. The NaCl deposit corresponds to small cubic crystals rich in Cl and Na, distributed as clusters on the corroded surface and amalgamated with the carbon-rich soots. Their low XRD signal (Figure 5) is due to the small size of the halite crystals.

Finally, note that the signal of tin is more attenuated than that of copper. This contrast may be explained by a deeper position of the tin in the patina or by a small proportion of Sn corrosion products localized on the surface but having an amorphous behavior and thus, undetected by XRD. Furthermore, traces of copper sulfate cannot be excluded (Section 3.1.2).

From the above, the topography of the alteration layer can therefore be considered as heterogeneous. On the one hand, a thin adherent layer, more or less micro-cracked (Figure 6b), is located in contact with the alloy and is composed of copper and tin corrosion products. On the other hand, clusters of calcite, gypsum or halite are deposited irregularly on its surface and do not cover it uniformly. This topographic heterogeneity results in a roughness that increases with the duration of alteration (Figure 6c) to reach a mean value of 19.2 µm ± 2.1 µm at the end of the tests. The evolution of the roughness (Sz) is correlated to the thickness of the particulate deposit and can be considered proportional to the average thickness of the final alteration layer [21]. However, the reactivity of the deposit with gases and relative humidity cannot be excluded [31].

#### 3.1.2. In-Depth Characterization of Bronze Specimens

**Figure 7:**
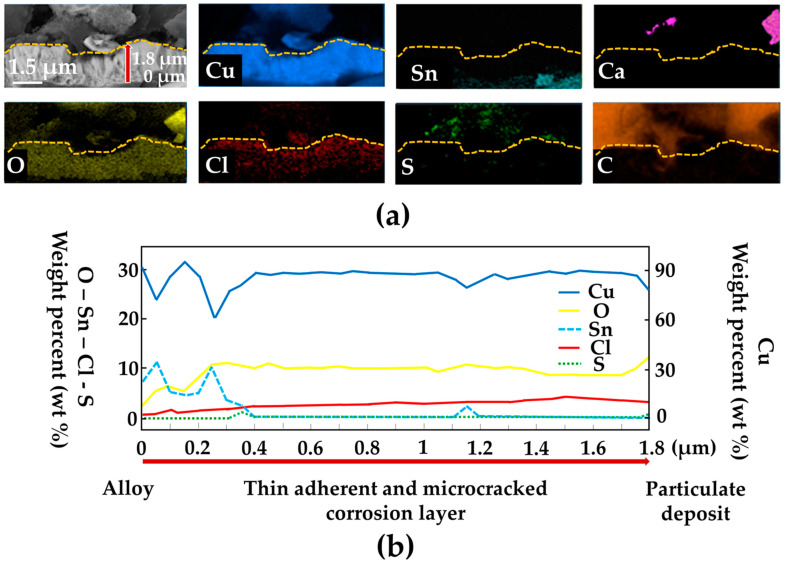
(**a**) SEM–EDS analysis and (**b**) composition of corrosion products in the lower part of the deposit determined by line analysis of the samples’ cross-section.

To better understand the internal organization of the corrosion layer, SEM-EDS cross-sectional analyzes of the corrosion layer were performed. Figure 7a shows the cross-section obtained after the third cycle. Its thickness does not exceed 4 µm because of the weak grip of the particulate clusters composed of calcite, gypsum and halite on the alloy substrate. This deposit was disturbed by the cross-sectional cutting. Thus, only the lower portion of the deposit is observable and consists of calcium (Ca) and carbon (C). The signal of sulfur is also detected and associated with that of copper (Cu), oxygen (O), carbon (C) and some traces of chlorine (Cl). The particulate deposit overlies a 1.8 μm thick layer, adherent to the alloy and composed of copper and tin corrosion products. A composition profile of this layer is shown in Figure 7b. The Cl signal, more present than in the upper deposit, is associated with homogeneous Cu and O signals. Locally, very fine traces of S are also detected. On the other hand, associated with a depletion in Cu, the Sn signal is present more in-depth and in contact with the alloy.

XPS analyzes with ion abrasion were performed to determine, in detail, the distribution of the corrosion products inside the patina. Therefore, this technique provides a better understanding of the physicochemical reactions between the alloy and the particulate deposit above, but also the alloy and the atmospheric gases propagating in the corrosion layer.

**Figure 8:**
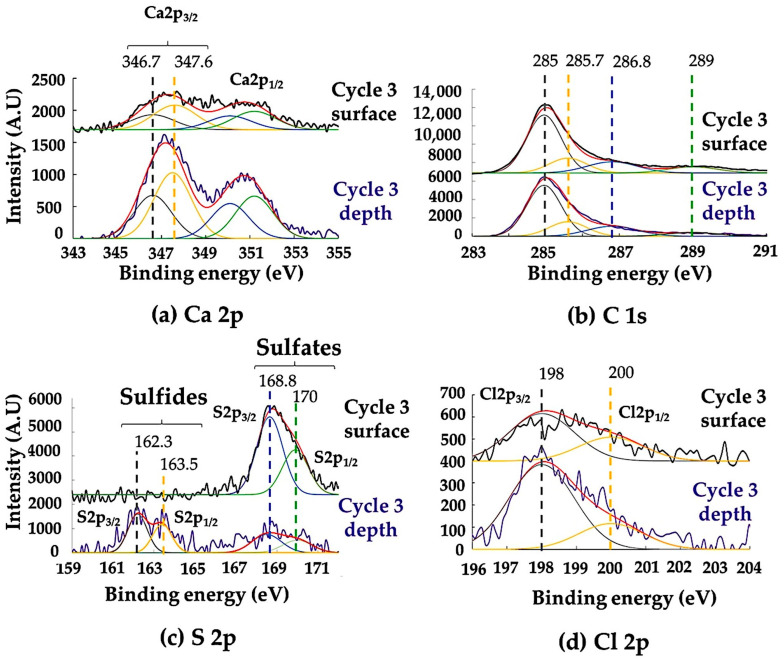
Deconvoluted XPS spectra of the surface and deep patina: (**a**) Ca 2p, (**b**) C 1s, (**c**) S 2p and (**d**) Cl 2p spectra.

**Figure 9:**
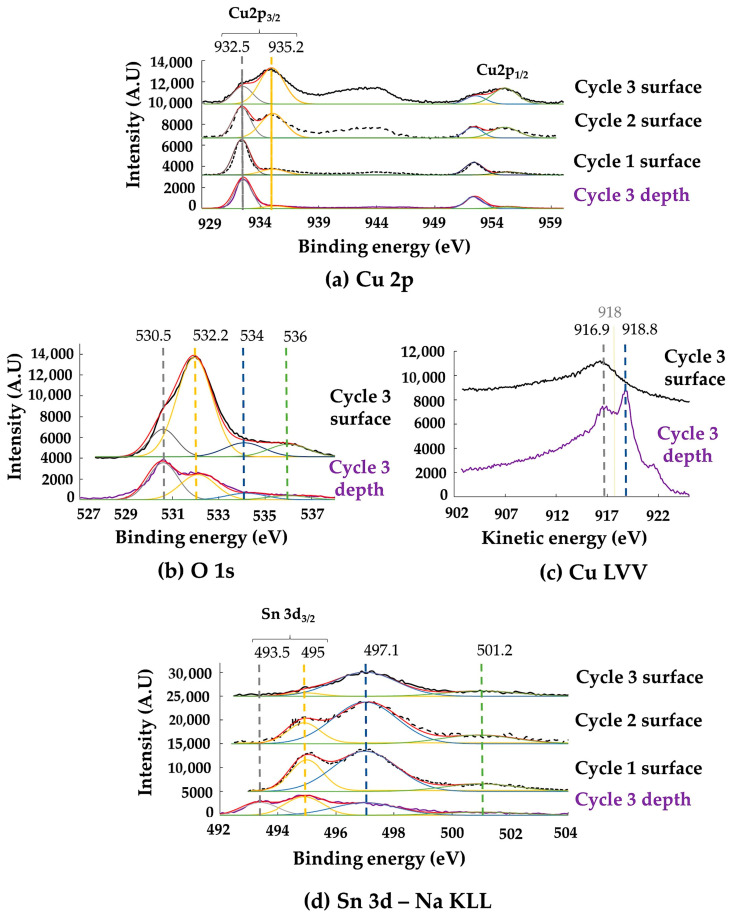
Deconvoluted XPS spectra of the surface and deep patina: (**a**) Cu 2p, (**b**) O 1s, (**c**) Cu LVV and (**d**) Sn 3d-Na KLL spectra.

The XPS analyzes are shown in Figure 8 and Figure 9. To study the effects of alteration duration, the spectra of each cycle, obtained at the extreme surface or after ion abrasion, are compared when significant qualitative or quantitative differences were observed. Otherwise, only the spectra of Cycle 3, characteristic of the longest alteration, are presented.

The XPS spectra show many broad peaks, indicating that many poorly crystallized constituents are present in the alteration layer.

Whichever the cycle studied, the binding energies of calcium Ca 2p, carbon C 1s, and sulfur S 2p (Figure 8a–d) confirm that the calcium of the particulate deposit is calcite (Ca 2p_3/2_ 346.7 eV, C 1s 289 eV) and gypsum (Ca 2p_3/2_ 347.6 eV, S 2p_3/2_ 168.8 eV) [32].

The presence of more or less hydrated soot is also confirmed. They consist of a graphite core (C 1s 185 eV) and organic compounds adsorbed on its surface (in the form of aliphatic carbon CH_2_-CH_3_ (C 1s 285.6 eV) and alcohol functions C-OH (C 1s 286.8 eV)) [33,34]. After ion abrasion, these species remain but sulfides S^2−^ (doublet S 2p_3/2_ 162.3 eV S 2p_1/2_ 163.5 eV) in the form of CaS_n_ and or CaS polysulfides are observed. This attribution is supported by the literature, in which previous studies reported that the Ca 2p_3/2_ peak of CaS_n_ is close to that of gypsum and that of CaS is expected at 346.45 eV [35]. Furthermore, the intensities of the Cl 2p_3/2_ and Cl 2p_1/2_ peaks increase after ion abrasion, which is characteristic of infiltration of marine aerosols into the alteration layer.

Regarding the copper corrosion products in the microcracked alteration layer, whichever the alteration cycle, the satellite peaks between 940 and 945 eV in the Cu 2p_3/2_ window correspond to the degree of copper oxidation Cu^2+^ (Figure 9a).

The Cu 2p_3/2_ peak at 935.2 eV and those of O 1s at 532.2 eV (O^2−^), 534–536 eV (OH^−^) (Figure 9b) associated with S 2p_3/2_ at 168.8 eV are characteristic of more or less hydrated copper sulfates in the form of brochantite, antlerite or chalcantite, where copper has an oxidation degree Cu^2+^ [36]. The intensity of the peak at 935.2 eV and of the satellites increases with the number of cycles, but they disappear with ion abrasion. The quantity of these copper sulfates increases with the duration of alteration but only constitutes a thin layer in contact with the alloy. Traces of copper carbonates CuCO_3_ (Cu^2+^) cannot be ruled out because of the proximity of the particulate clusters of soot and carbonates. A Cu-2p_3/2_ peak assigned at 932.5 eV, specific to a Cu^+^ oxidation degree, is also present. Associated with the O 1s peak at 530.5 eV, it corresponds to cuprite Cu_2_O (Cu^+^). Contrary to the Cu 2p_3/2_ peak at 935.2 eV, the one at 932.5 eV persists with the stripping. However, the 2p spectra of copper metal Cu and cuprite Cu_2_O are difficult to differentiate because of their similar morphologies and very close binding energies (±0.2 eV). To differentiate Cu and Cu_2_O, it is common to refer to the Auger AES-LVV spectrum, in which shifts between the oxides and copper are larger (Figure 9c). The shoulder of the LVV peak at a kinetic energy of approximately 917 eV denotes the presence of in-depth Cu_2_O. Conversely, the non-detection of a peak at 918 eV confirms the absence of CuO [37].

Concerning the tin corrosion products (Figure 9d), the Sn 3d_3/2_ peaks at 495 eV and O 1s are at 495 eV and 530.5 eV, respectively. Since the chemical and spectral separation of tin oxides (SnO (Sn^2+^) and SnO_2_ (Sn^4+^)) is difficult when only based on their core XPS spectra [38,39], we followed [40,41] and assumed that the Sn peaks are relative to the Sn^4+^ species (SnO_2_ tenorite). In the absence of abrasion, the Sn 3d_3/2_ peak at 495 eV decreases in intensity after three cycles of alteration. The copper corrosion products close to the surface act as a shield and decrease the intensity of the signal of the tenorite located deeper, in contact with the alloy. With ion abrasion, the Sn 3d_3/2_ peak at 493 eV of metallic tin appears, associated with that of SnO_2_ at 495 eV. The width of the S 2p_3/2_–S 2p_1/2_ doublet from 161 eV to 164.5 eV, characteristic of sulfides (S^2−^), and the Sn 3d_3/2_ peaks at 495 eV (Sn^4+^) and Cu 2p_3/2_ at 530.5 eV (Cu^+^) cannot exclude traces of deep copper or tin sulfides [42,43].

Finally, the Auger KLL of Na (497.1 eV, 501.2eV), located in the same window as Sn 3d_3/2_ and characteristic of NaCl, decreases with stripping. This is not the case of chlorine (Figure 8d). This difference confirms the infiltration of marine aerosols, but not in the form of halite. Their dissolution releases Cl^−^ ions that infiltrate the particulate deposit and react with Cu to form deep CuCl nantokite, as evidenced by the Bragg Brentano XRD. No trace of copper hydroxychloride CuCl_2_(OH)_3_ is detected (absence of the Cu 2p_3/2_ peak at 937 eV [44]).

### 3.2. Effect of the Corrosion on the Acoustic Properties of Bronze Samples

**Figure 10:**
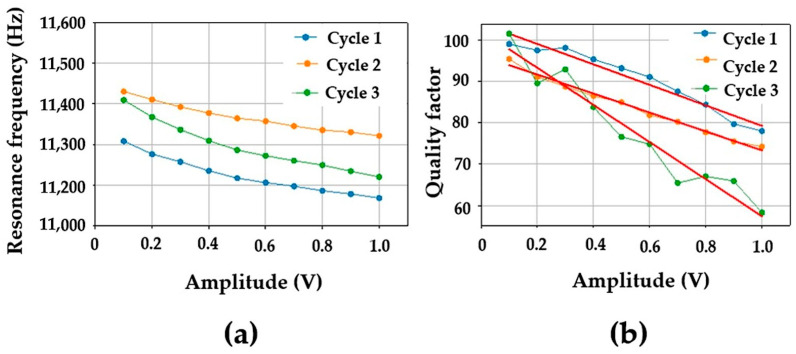
Evolution of the frequency (**a**) and the quality factor versus the excitation amplitude (**b**) corresponding to the 4th bending resonance as a function of the excitation level. The exposure times are: cycle 1 = 1 month, cycle 2 = 2 months and cycle 3 = 3 months.

Based on the experimental set-up presented above, nonlinear resonance experiments are performed in bending conditions at increasing excitation levels as described above. Results presented in Figure 10 show that the corrosion cycles create a softening in the elastic modulus. The softening is observed through the decrease in the resonance frequency when the excitation amplitude is increasing (the real part of the elastic modulus increases (or decreases) with increasing (or decreasing) resonance frequency). However, the decrease is not evolving in a monotonous way with the cycles applied. Indeed, at the weakest excitation level, the bending resonance frequency is the highest for Cycle 2 and the weakest for Cycle 1. Cycle 3 is found to be always between Cycles 1 and 2. On the other hand, the frequency drop due to an increase in excitation for each cycle is found to be ~120 Hz, ~110 Hz and ~190 Hz for Cycles 1, 2 and 3, respectively. This result shows that regardless of the linear resonance frequency value (obtained at a weak level), the frequency drop between the weakest and the highest excitations can be used as an indicator to monitor the corrosion evolution. In addition, results related to the quality factor, which is inversely proportional to damping, show that the three cycles have almost the same value at the weakest excitation level. The slope of their evolution (see Table 2) with an increasing excitation shows that Cycles 1 and 2 have almost the same slopes with P_1_ = −24.7 × 10^−3^ (v^−1^) and P_2_ = −22.84 × 10^−3^ (v^−1^), respectively. The slope for Cycle 3 is found to be P_3_ = −44.80 × 10^−3^. This value, which is twice that found for Cycles 1 and 2, shows again the sensitivity of the nonlinear vibration method to the structural evolutions created by corrosion.

The study of the second-order effects related to the complex elastic modulus, through the resonance frequency and the quality factor, showed that the observed changes in the resonance frequency and the slope of the quality factor can double in just 1 month, between Cycles 3 and 2. It is important to recall that the deterioration concerns only one side of the samples studied. The corrosion depth being small had mainly affected the tested specimens superficially, attesting to the absence of a bulk effect on the elastic properties. This is the reason why the recorded nonlinear responses for Cycles 1 and 2 were similar, contrary to Cycle 3, for which a better sensitivity was found. Note that, at the initial intact state, neither frequency nor quality factors have changed as a function of the excitation.

## 4. Discussion

Previous studies of the atmospheric alteration of bell bronze had shown a microinfiltrative property of the patina and the development of preferential α/δ corrosion of the underlying alloy [10,11]. They were performed on samples corroded over a long term. In order to better understand the early stages of corrosion and its undocumented impacts on the acoustic properties of the alloy, the physicochemical alteration was reproduced in the laboratory under realistic conditions. More precisely, a polluted marine environment with no direct rainfall, which corresponds to the sheltered configuration of steeples, was simulated.

**Figure 11:**
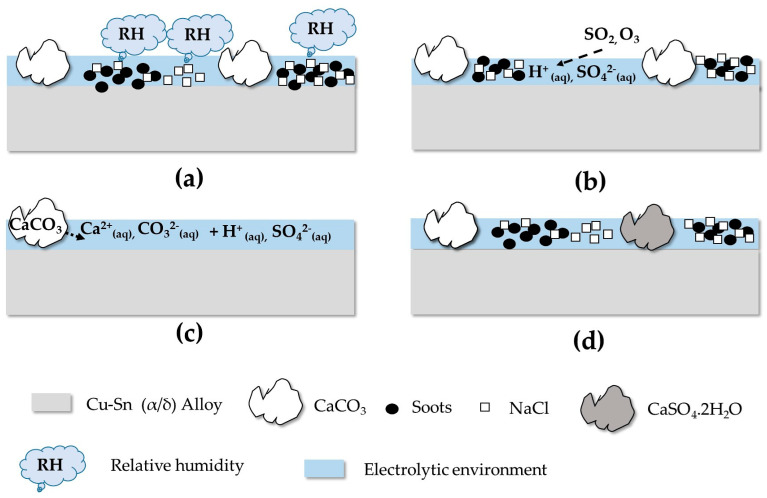
Diagram of the different reaction steps (**a**–**d**) between the atmosphere in the CIME chamber and the particulate deposit.

**Figure 12:**
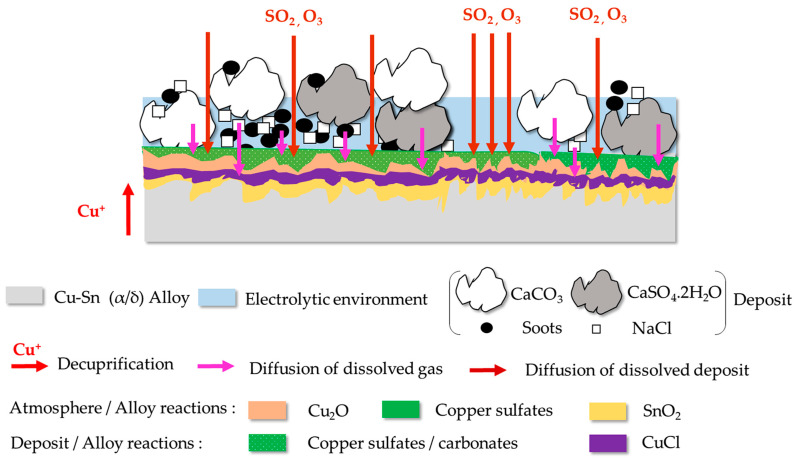
Diagram of the alloy reaction with the atmosphere in the CIME chamber and the particulate deposit.

The experimental results confirm the complexity of the physicochemical alteration. In contact with the alloy, a thin, adherent and micro-cracked layer formed. It was mainly composed of sulfates, copper oxide and chloride overlying other corrosion products rich in tin. On the surface, less adherent irregular clusters of soot, calcite, gypsum and halite developed. Based on these observations, an alteration scenario corresponding to a corrosion mechanism can be proposed, corresponding to three distinct reactions illustrated in Figure 11 and Figure 12.

(1) Reaction between the atmosphere of the CIME chamber and the particulate deposit:

Soot and halite particles are known to have hygroscopic properties [45,46,47]. They tend to absorb ambient humidity to form an electrolytic environment where the anthropic gases SO_2_ and O_3_ can dissolve and react with the particulate clusters. Calcite is the most reactive element when in contact with sulfur dioxide and ozone. Indeed, a high concentration (600 ppb) of O_3_ injected into the CIME chamber is sufficient to oxidize SO_2_ to SO_3_ which, dissolved by the condensation water on the surface of the deposit, leads to the formation of H_2_SO_4_ [48]. This one reacts with Ca^2+^ ions from the dissolution of calcite to form gypsum [49].

According to the XPS and DRX investigations, sulfur penetrates inside the deposit where calcium sulfides and sulfates were detected. Their formation results from the diffusion of SO_2_, O_3_ and H_2_SO_4_ or from the dissolution of part of the gypsum. These results underline the micro-infiltrative and hygroscopic character of the particulate clusters with respect to humidity and anthropic gases that accelerate the underlying metallic corrosion [6,8] and the formation of insoluble corrosion products of the bronze [50].

(2) Reactions between the atmosphere and the alloy:

The atmospheric conditions reproduced in the CIME chamber (400 ppb SO_2_, 600 ppb O_3_) are corrosive enough to interact with the alloy underlying the deposit and form cuprite and copper sulfates. On the bronze surface, the particulate deposit is non-uniform. The electrolyte forms by condensation on the surface of the hygroscopic deposit and migrates towards the underlying alloy, or it condenses on contact with the alloy in the parts deprived of the deposit. The dissolved oxygen in the electrolyte causes thermodynamic instability of copper: Cu^+^ cations are emitted (decuprification) and combine with oxygen to form a cuprite layer adhering to the alloy, near and under the particulate deposit. Cu_2_O is one of the first corrosion products to appear [51]. For the copper sulfates, they come from the combination of SO_4_^2−^ from the SO_2_ dissolved in the electrolyte and Cu^2+^ from the oxidation of Cu^+^ at the cuprite/electrolyte interface. Concerning the tin alteration, the corrosion products are in contact with the alloy, under the cuprite layer and as SnO_2_ tin oxide. Traces of tin and copper sulfides cannot be excluded.

(3) Reactions between the deposit and the alloy:

The alloy interacts with the microfiltration deposit that covers it. By dissolving, the gypsum, the soot and calcite are, respectively, a source of SO_4_^2−^ and CO_3_^2−^ ions, which migrate through the deposit and react with copper ions to form under deposit amorphous copper carbonate CuCO_3_ associated with copper sulfates on the surface of the cuprite. The copper sulfates have then a dual origin: a reaction between the atmosphere and the alloy but also a reaction between the deposit and the alloy. Concerning the NaCl deposit, it also dissolves and emits chloride ions to form nantokite (CuCl). As compared to copper sulfates, it occupies a deeper position in the alteration layer. This reflects a greater penetration of chlorine than sulfur through the deposit. CuCl, commonly observed in contact with the alloy, is the start of the so-called bronze disease characterized by the development of copper trihydroxychloride CuCl_2_(OH)_3_ [52]. It requires significant inputs of water and oxygen which are not reached in our alteration conditions because no trace of CuCl_2_(OH)_3_ is detected.

The depth of tin corrosion products is characteristic of Type I corrosion, typical of low-corrosive environments for Cu-Sn alloys [4]. Despite the micro-infiltration character of the particulate deposit, which allows the diffusion of sulfur, oxygen and chlorine toward the alloy, the corrosion kinetics of the underlying bronze is controlled by a decuprification phenomenon. Copper ions diffuse through the tin-rich layer (passivation properties) before reacting with anions in the deposit and dissolved gases released by the chamber atmosphere. The amount of cuprite, copper sulfates and carbonates increases with the duration of alteration, as well as the gypsum in the deposit. As far as our investigations go, no preferential α/δ corrosion of the bell alloy was observed. This result would tend (1) towards further development of this preferential corrosion, observed on long-term corroded bells [10] and (2) question the role of metal inclusions and microcracks of the alloy in its development.

On the other hand, the excited vibration in the nonlinear regime is shown to be sensitive to the small changes induced by corrosion. Indeed, the development of the corrosion layer, which corresponds to a surface alteration of the bronze, causes a deterioration of its mechanical properties (density and the complex elastic modulus). The registered softening is due to the dependence of the mechanical properties on the dynamic strain, which in general does not exceed 10^−5^. Furthermore, the presence of the corrosion layer is also responsible for the fast enhancement of the dissipative properties, i.e., absorption of the bronze. This deterioration in the acoustic properties, which are directly linked to the mechanical properties, appears and can be detected through the overall behavior of the samples tested as soon as a very thin corrosion layer develops. At this stage of the study, we have shown that the sensitivity of the physical properties, corresponding to the corrosion layer to different parameters, such as the presence of microcracks in contact with the alloy, the extreme external surface, the poorly adhering and non-compact particulate deposit, is at the origin of its acoustic nonlinear behavior. However, the sensitivity of the nonlinear behavior to the presence of corrosion could be improved by considering transient elastic waves. Indeed, the propagation of the latter, for wavelengths much smaller than the ones of the standing waves, will improve the interaction with the small local variations due to corrosion such as localized microcracks or severe surface roughness on the hidden side of the bell [53].

## 5. Conclusions

This work has allowed us to reproduce in the laboratory, under realistic conditions, the alteration of the bell bronze in a polluted marine atmosphere and in the absence of direct rainfall.

The corrosion layer consists of two parts:

(1) A thin adherent layer, more or less microcracked, is located in contact with the alloy and is composed of copper and tin corrosion products: sulfates, copper oxide and chloride cover corrosion products rich in tin oxide.
(2) Clusters of soots, calcite, gypsum and halite are irregularly distributed on its surface and do not cover it uniformly.

This alteration is complex and must take into account the joint interactions between the alloy, the particulate deposit and the atmospheric gases, but also the microinfiltration and hygroscopic properties of the corrosion layer in the formation. A Type I mechanism dominates. Despite this complexity, combined with the small size of the existing features, nonlinear acoustic resonance measurements have shown good sensitivity to the changes induced by corrosion.

The deterioration of the mechanical properties has been detected through the softening of the elastic modulus, which can be considered as a homogenization between the one corresponding to a major part of the thickness that remained intact and modulus of the thin corroded zone.

However, there were limitations or challenges encountered during this study.

(1) Thanks to the limited amount of porosity and microcracks in our corpus of uncorroded bronze samples, we can objectively validate the objectives of our study, namely a better understanding of atmospheric corrosion of bronze used in bell making and its effects on the tonal performance of bell bronzes. In the present work, the specimens did not undergo any mechanical alteration. However, as soon as they are installed, bells undergo a double alteration: chemical as a result of the progressive corrosion by the atmosphere, but also mechanical, due to the repeated action of the clapper or the hammer used to make them ring [18]. Over time, cracks may form on the bell as a result of its use and this mechanical damage is known to be a key factor in the modification of the bell’s tones [18,19].
(2) The study presented in this article was performed on parallelepiped bronze specimens. We have not taken into account the role of the typical forms of the bells.

Therefore, additional investigations and experiments should be conducted to further deepen our understanding of the relationship between atmospheric corrosion and the sound quality of bronze bells:

(1) Beyond the experimental results related to the nonlinear parameters (resonance frequency and quality factor), other results based on guided waves can also be obtained. Indeed, by considering higher frequencies in the range of megahertz, surface Rayleigh waves can be generated in the nonlinear regime in order to optimize the interaction with corrosion. Finally, Lamb waves (which are guided by the thickness of the sample) can also be generated in linear and nonlinear dynamic conditions, where the sensitivity of these dispersive waves to the change of thickness can be used as an indicator [54,55].
(2) A comparative study of corrosion reproduced in the laboratory on microcracked bronze specimens will enable us to determine the impact of mechanical damage on corrosion mechanisms and the acoustic properties of corroded bronze.
(3) The sound of bell is a sum of modal contributions [56,57,58]. The impact of the corrosion reproduced in the laboratory on the sound of a bell will be evaluated by estimating the damping and distortion of the bell modes in the audible range of the frequencies. Experimental modal analysis of bells, carried out, for example, using a high-resolution method [59], can be performed by impact hammer tests and accelerometer or vibrometric measurements.

This research, dedicated to a better understanding of the atmospheric corrosion of bell bronze and its impacts on its sound properties, will ultimately contribute to better preservation of this material but also immaterial sound heritage, in the same way as the taste and smells of the past [60]. Indeed, a better understanding of the origin of the sound deterioration of bell bronze will contribute to enriching research dedicated to the restoration of the soundscape of the past, as well as the preservation of the original sound of these musical instruments. This is advocated today by many organizations such as the French Society of Campanology (SFC) and the European Conservatory of Bells and Clocks of Edifice (CECH). The results of this study can also be applied to other metal percussion instruments, such as cymbals and gongs [61,62].

## Figures and Tables

**Table 1:** Average chemical composition of each of the 5 uncorroded bronze specimens in weight percent (wt%) and the average chemical composition (wt%) of the 5 uncorroded bronze specimens.

	Cu	Sn	Zn	Pb	Ni	Mn	P	Sb	Al
Sample 1	76.210 ± 0.780	22.210 ± 0.340	0.02 ± 0.004	0.020 ± 0.005	0.007 ± 0.0012	0.008 ± 0.002	0.006 ± 0.001	0.008 ± 0.001	0.009 ± 0.003
Sample 2	77.370 ± 0.970	21.610 ± 0.190	0.03 ± 0.006	0.020 ± 0.003	0.01 ± 0.002	0.007 ± 0.002	0.008 ± 0.001	0.007 ± 0.002	0.010 ± 0.001
Sample 3	78.540 ± 0.770	21.680 ± 0.270	0.025 ± 0.002	0.015 ± 0.004	0.003 ± 0.001	0.003 ± 0.001	0.007 ± 0.002	0.001 ± 0.001	0.003 ± 0.001
Sample 4	79.35 ± 0.320	21.340 ± 0.203	0.010 ± 0.003	0.012 ± 0.003	0.002 ± 0.001	0.002 ± 0.001	0.005 ± 0.002	0.002 ± 0.001	0.001 ± 0.001
Sample 5	79.800 ± 0.890	21.880 ± 0.380	0.01 ± 0.003	0.002 ± 0.001	0.001 ± 0.001	0.002 ± 0.001	0.002 ± 0.001	0.002 ± 0.001	0.002 ± 0.001
Samples 1–5	78.254 ± 1.171	21.744 ± 0.241	0.019 ± 0.007	0.014 ± 0.005	0.005 ± 0.003	0.004 ± 0.002	0.006 ± 0.002	0.004 ± 0.002	0.005 ± 0.003

**Table 2:** Slopes of the quality factor evolution as a function of the excitation level.

Type of Chamber	Exposure Time	Slope (v^−1^)	Linear Regression Coefficient (R^2^)
CIME	Cycle 1 (1 month)	−24.71 × 10^−3^	0.976
	Cycle 2 (2 months)	−22.84 × 10^−3^	0.995
	Cycle 3 (3 months)	−44.80 × 10^−3^	0.971

## Data Availability

All required data provided in the manuscript. No new data were created or analyzed in this study. Data sharing is not applicable to this article.
